# The study protocol for a pseudo-randomised pre-post designed controlled intervention trial to study the effects of a 7-week cooking program on self-efficacy and biomarkers of health: the ECU lifestyle and biomarkers get connected study (ECULABJMOF) including the Jamie’s Ministry of Food WA participant experience

**DOI:** 10.1186/s12889-020-09124-3

**Published:** 2020-06-30

**Authors:** Joanna Rees, Claus C. Christophersen, Joshua R. Lewis, Johnny Lo, Ros Sambell, Leesa Costello, Cailyn Walker, Matt F. Byrne, Mary C. Boyce, Robert U. Newton, Amanda Devine

**Affiliations:** 1 School of Medical and Health Sciences, Edith Cowan University, 270 Joondalup Drive, Joondalup, Perth, WA 6027 Australia; 2 WA Human Microbiome Collaboration Centre, School of Molecular & Life Sciences, Curtin University, Perth, WA Australia; 3 Medical School, University of Western Australia, Perth, Australia; 4 School of Public Health, University of Sydney, Sydney, Australia; 5 School of Science, Edith Cowan University, Perth, WA Australia; 6 School of Education, Edith Cowan University, Perth, WA Australia; 7 Centre for Integrated Metabolomics and Computational Biology, Edith Cowan University, Perth, WA Australia; 8 Exercise Medicine Research Institute, Edith Cowan University, Perth, WA Australia; 9 School of Human Movement and Nutrition Sciences, University of Queensland, Brisbane, QLD Australia

**Keywords:** Cooking program, Self-efficacy, Dietary intake, Microbiota, Mental health, Study protocol

## Abstract

**Background:**

Australia, like other nations, has experienced a shift in dietary patterns away from home cooking of nutritious foods, towards a reliance on pre-prepared convenience meals. These are typically energy-dense, nutrient-poor and contribute to the rising prevalence of obesity and chronic disease burden. The aims of this study were to evaluate whether a community-based cooking program instigated a change to participants’ skills, attitudes, knowledge, enjoyment and satisfaction of cooking and cooking confidence (self-efficacy).

**Methods:**

The pseudo-random, pre-post study design consisted of an intervention and a control group. Participant recruitment and group allocation was based on their program start dates. Intervention participants were surveyed three times (baseline, 7 weeks and 6 months) and the control group were surveyed at baseline and 5 weeks. All participants were registered via an online website and were 18 years or over. Upon consent, participants were offered four levels of commitment, defined by different assessments. The minimum participation level included an online survey and levels 2, 3 and 4 involved attendance at a clinic with increasing functional, anthropometric and biomarker measurements. Primary endpoints were participants’ cooking confidence as a proxy for self-efficacy. Secondary endpoints were dietary intake, physical activity levels, body composition, anthropometry, blood, urine and faecal biomarkers of systemic, physical and mental health.

**Discussion:**

The community cooking program provided participants with information and advice on food sourcing, preparation and nutrition to improve home cooking skills. The study was designed to explore whether food literacy programs are efficacious in improving participant physical health and well-being in order to combat the rise in obesity and diet-related disease**.** It will support future use of public health cooking program initiatives aimed at improving food literacy, self-efficacy and physical and mental health. The extensive data collected will inform future research into the relationship between diet, the gut-microbiota and human health.

**Trial registration:**

Retrospectively registered on 16.08.2019 with the Australian New Zealand Clinical Trials Registry (ANZCTR). ACTRN12619001144101.

Protocol version 4.

## Background

Healthy eating patterns and good food choices are essential for promoting health and well-being and preventing a wide range of chronic diseases [1], such as cardiovascular disease [2], Type 2 diabetes [3], some cancers [4] and obesity [5]. Yet over the past few decades, Australia like most other nations has experienced a dramatic shift in daily dietary patterns away from preparing and cooking nutritious home food, towards a significant reliance on pre-packaged, prepared, convenience meals [6, 7]. This shift is reflected by current statistics showing that those with insufficient fruit and vegetable intake has risen by 8.5% for men and 10.8% for women since 2004/5 [8]. The increasing demands of today’s modern lifestyles appear to limit the amount of time spent on home cooking. In response, the food industry has grown substantially, offering fast food and ready-made meals which are often energy-dense, nutrient-poor, with high levels of salt, saturated fat and sugar [9].

The Australian Bureau of Statistics (ABS) National Health Survey (2017–18) [10] showed 67.0% of Australian adults were overweight or obese (12.5 million people), an increase from 63.4% in 2014, placing a considerable burden on the healthcare budget and economic growth [11]. In addition, mental health and behavioural conditions affect 20% of the Australian population [10]. Epidemiological studies have found that depressive disorders are closely linked to cardiovascular and metabolic health outcomes and share a similar aetiology [12–14]. There is overwhelming evidence that a transition towards a Westernised diet high in processed foods, fats and protein and low in fresh fruit, vegetables and dietary fibre (DF) is associated with higher rates of both metabolic disease and mental health disorders [15–17]. Further the effect of a Western diet on the gut microbiome has been reported to induce dysbiosis, that is related to a compromised metabolic profile in the gut, which impacts mental and physiological health [18, 19].

Social determinants of health such as socio-economic status and household income are associated with food purchasing decisions and dietary intake, whereby households of lower income and those in rural and remote areas often have poor diet quality and greater risk of disease [20–22]. Barriers such as the price of fresh produce and lack of cooking skills can influence purchasing behaviours for healthier foods, especially among lower socio-economic families and those who are food insecure [23–25]. Therefore providing individuals, families and communities with food literacy and access to information to develop budgeting skills, efficient food shopping strategies, and skills to prepare healthy meals at low cost, may overcome economic barriers that presently prevent healthy eating [26, 27]. The proliferation of community-based cooking interventions to address these issues is well documented [23, 24, 28–32] and interest has been stimulated by media attention and prime time cooking programs with celebrity chefs [33]. However, to date the evidence of efficacy has been based on small-scale evaluations with methodological limitations [24, 34]. During the lead-up to this study, various systematic reviews of community cooking interventions reported that, although most observed positive changes in cooking confidence, findings were inconclusive due to a lack of quality evaluation methods and small convenience samples with female predominance [35–37]. A later systematic review [30], (published after the commencement of this study), further emphasised the need for future intervention studies to include a control group, follow-up beyond program completion and the use of validated assessment instruments. Reicks et al. [30] also reported that there was still a limited number of studies that included clinical measures of health and lifestyle.

These reviews emphasise the need for more rigorous, large-scale longitudinal studies to examine the range of impacts and outcomes of cooking skill programs and underlying factors that influence behavioural change [34] and that include potential drivers that are influenced by health, taste, cost, time, convenience, family responsibilities, familiarity and confidence [38–41]. The Jamie’s Ministry of Food (JMOF) Australia was a community-based program that commenced in 2016 in Western Australia (WA), teaching basic cooking skills to help people prepare simple, fresh, healthy food quickly and cheaply. It therefore provided an excellent opportunity for a longitudinal study to examine the physiological benefits of food literacy and cooking programs and to evaluate their sustainability over time. This paper outlines the study protocol for the quantitative evaluation of the JMOF cooking program, it outlines participant recruitment, collection of data and proposed analysis of food literacy, dietary intake, physiological measurements and biomarkers of health.

JMOF was brought to communities across Australia by The Good Foundation (TGF). In Western Australia (WA) the program was delivered by TGF and supported by Edith Cowan University (ECU) as part of a three-year partnership. Through the use of a mobile kitchen, the program was delivered to communities across the state and involved a series of hands-on, basic, 7-week cooking classes led by a team of dedicated and nutritionally qualified Food Trainers who were supported by local volunteer ECU Student Interns. The cooking course was open to people aged 12 years and over, looking to learn the basics of cooking while having some fun in the kitchen with Jamie’s hints, tips and shortcuts. To date there have been two previous evaluations of the program that were designed by [42–45] and included the online questionnaire that is used in this study. It featured questions specific to the JMOF program logic model [42] and was referred to in a subsequent peer review publication of JMOF evaluation by Herbert et al. [45]. The questionnaire contained the Rosenberg Self-Esteem Scale, which is a widely used and validated tool [46]. Standard evaluation information, consent, demographic details and participant contact details were also collected via the questionnaire. The underlying design of this study and the development of the questionnaire were based on the logic models described by Flego et al. [42] and were the property of the TGF. ECU collaborated with TGF to build on previous evaluation studies by including measurements of lifestyle and biomarkers of health to further explore the benefits of the program, however the basic design was limited to confines of the previous evaluations.

The aims of this study were to evaluate whether the JMOF program instigated a change to participants’ skills, attitudes, knowledge, enjoyment and satisfaction of cooking and cooking confidence (self-efficacy). This aim was based on the premise that preventive nutrition, as delivered by a program such as the JMOF program, along with healthy connections to others, would improve quality of life and health outcomes with an associated minimisation of chronic disease risk factors that that would be sustained over time.

The primary evaluation explored cooking confidence (operationalised as personal beliefs of self-efficacy) and the ability to cook a meal from healthy ingredients. The secondary evaluation sought to determine whether improvements to self-efficacy influenced lifestyle and dietary intake and other underlying mechanisms that led to positive physiological and mental health and well-being, measured by biomarkers of chronic disease risk.

The study hypothesised that participation in a program to develop basic cooking skills and nutrition knowledge would improve cooking confidence (self-efficacy), that would impact lifestyle to improve dietary intake, thereby having a positive influence on physiological and mental health and their biomarkers.

## Methods

### Study design

**Fig. 1:**
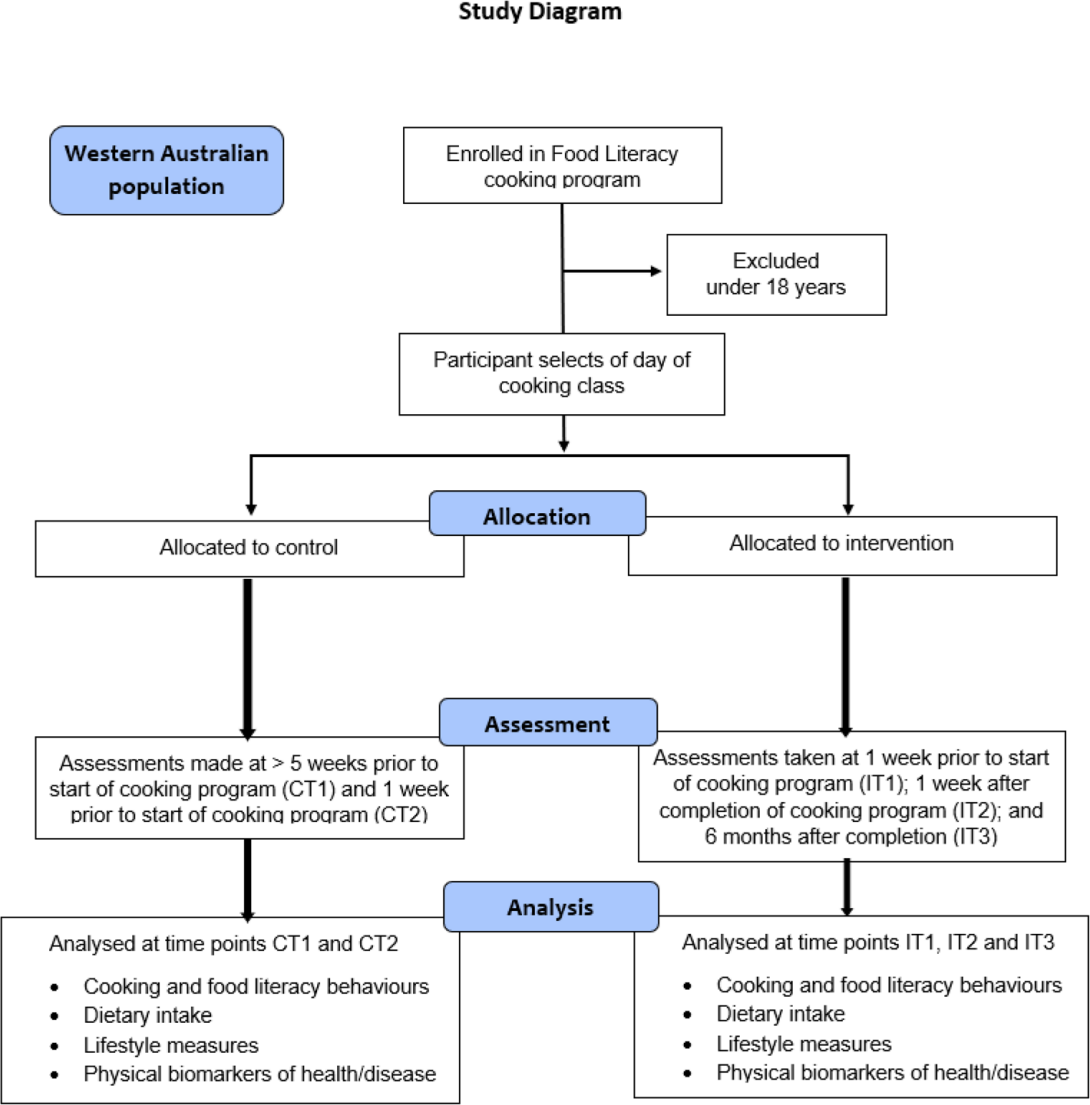
Overview of the study design

The study has a pseudo-random, mixed-methods, pre-post design involving an intervention group of participants who completed a cooking program that consisted of a 1.5-h session once a week for 7 weeks, in eight locations throughout WA. There was also a control group that comprised participants from these locations who were on the program wait-list (Fig. 1).

The study design was also premised upon the idea that participants would receive a continuity of support and education after attending the JMOF program. The qualitative component of the study incorporated formative research to determine what type of communication platform this could be delivered through. It involved interviews and focus groups with participants during the first round of the program who were asked about their program experiences, which provided insights that helped to explain the primary endpoints, as well as any impacts not captured by the quantitative measures. The outcome of which led to the development of a Facebook group that future participants could join, share their experiences and access educational links. Results from the qualitative components of this study have been reported but not published.

### Study participants

Participants were selected from those enrolled in the cooking program who were community-dwelling adults, 18 years and above, who volunteered to participate in the evaluation of the cooking program; no other inclusion or exclusion criteria were applied. Ethics approval was provided by the ECU Human Research Ethics Committee (HREC) (ID 15362:Newton). All participants gave their written consent prior to commencement in the study.

### Recruitment strategy

The study used a convenience sample of all participants 18 years and above, who registered for the JMOF program during the 3 years of its operation. A total of 19 courses were offered at various designated locations in WA over the 3 years and recruitment for these was conducted from 17/05/16 to 1/11/18. Each course was promoted via a portal on the JMOF website, firstly to the local community 3 months prior and secondly to the public 2 months prior to the designated start date. (https://www.jamiesministryoffood.com.au/courses). Registration for each cooking program became available 6 weeks prior to its commencement. Metropolitan courses were held in the suburbs of Joondalup, Mt Lawley, Belmont, Mandurah, Sienna Wood and Baldivis and regional courses were held in Bunbury and Albany in South West WA. TGF managed the Australian website and the registration database for all courses. Reports of WA program registrants were sent to ECU Survey Research Centre (SRC) who contacted eligible adult participants to determine their interest to be involved in the study and the level at which they chose to participate. For both control and intervention groups there were four levels of contribution, each building on the previous one by the inclusion of additional testing (Table 1). Those who opted into the study received a link to an online consent form and evaluation survey. Withdrawal was an option at any stage or level of participation.

**Table 1:** Participation level

Level	Online questionnaire (includes, SF-12, SVS, WEMWBS, IPAQ, GLTEQ, and beverage intake)	FFQ (DQES v2)	Anthropometry, blood pressure, body composition, mobility, agility and strength	Blood and urine samples (Lipids, glucose, *hs-*CRP, IL-6, LPS, carotenoids,	Stool samples (SCFA, BA, microbiome)
1 – Bronze	**√**	**×**	**×**	**×**	**×**
2 – Silver	**√**	**√**	**√**	**×**	**×**
3 – Gold	**√**	**√**	**√**	**√**	**×**
4 – Platinum	**√**	**√**	**√**	**√**	**√**

(*SF-12* The 12-item Short Form Survey, *SVS* Subjective Vitality Scale, *WEMWBS* Warwick-Edinburgh Mental Well-being Scale, *IPAQ* International Physical Activity Questionnaires, *GLETQ* Godin Leisure-Time Exercise Questionnaires, *DQES FFQ* Dietary Questionnaire for Epidemiological Studies Version 2 Food Frequency Questionnaire, *hs-CRP* High sensitivity C-Reactive Protein, *IL-6* Interlukin-6, *LPS* Lipopolysaccharide, *SCFA* Short Chain Fatty Acids, *BA* Bile Acids)

### Assignment of groups

Subsequent sampling for the control and intervention groups was purposive from the program registration database. Classes were offered on all days of the week except Sunday and the day chosen by the participant determined their allocation to either the control group or the intervention group. If a subject met the inclusion criteria of being 18 years and above and selected a class on either a Monday or a Thursday, they were eligible to become a control participant. Intervention participants were those who met the inclusion criteria of being 18 years and above and selected a class on any of the other days of the week. This sampling approach and allocation of registered people to the two groups was, therefore, pseudo-random and chosen to fit in with the design and philosophy of the program, which emphasises the importance of participants attending the program when and with whom they want.

#### Intervention

**Fig. 2:**
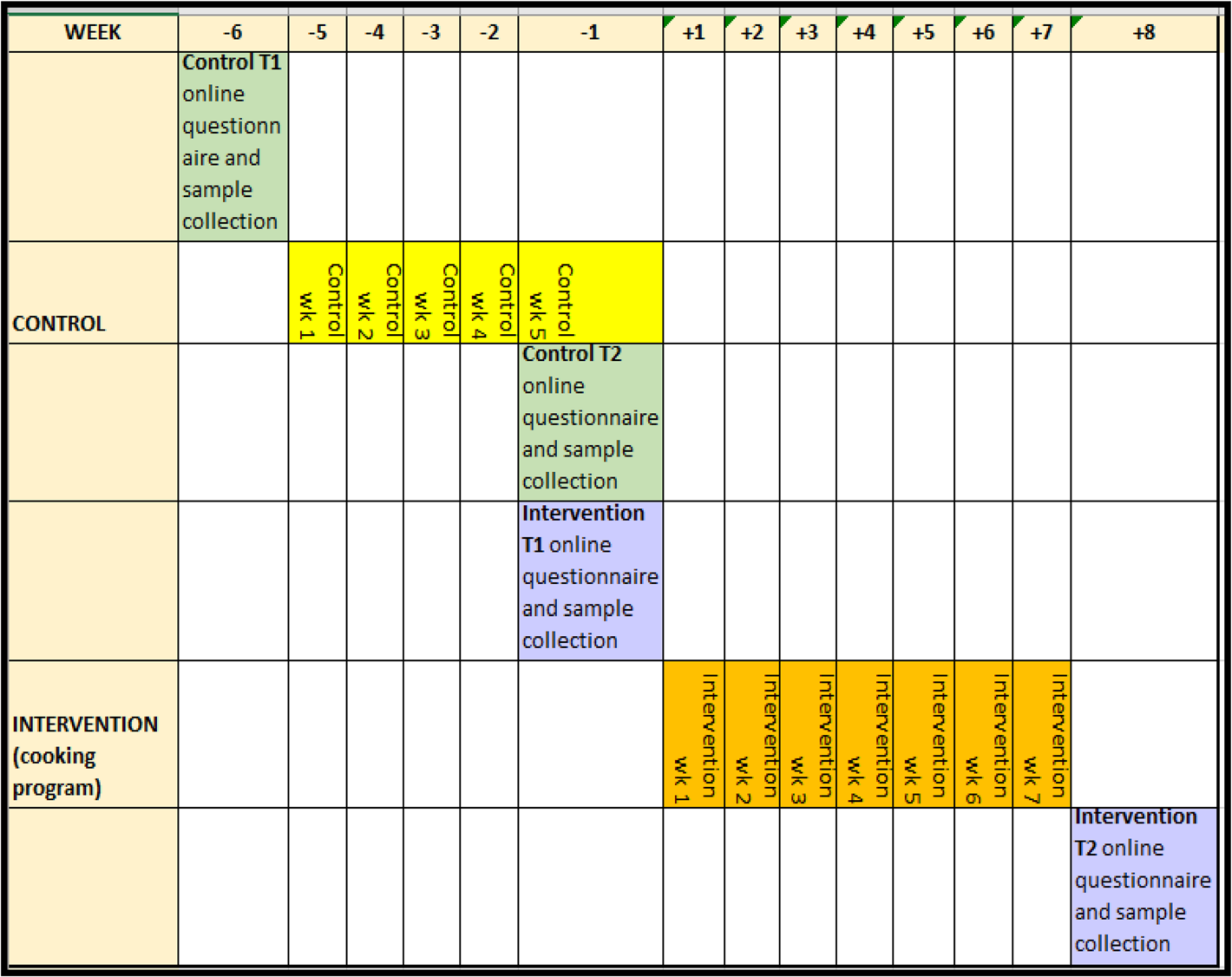
Study timeline

The intervention involved participation in one of the nineteen 7-week cooking programs that were delivered at six metro and two regional locations throughout WA from the fully equipped JMOF mobile kitchen. Each program involved a 1½ hour hands-on cooking session held once per week for 7 weeks. The weekly sessions were scaffolded in their recipe complexity to enable increased cooking confidence, skill development and food literacy knowledge. Classes were delivered by a JMOF-trained, qualified nutritionist who demonstrated the recipe of the day which participants then cooked for themselves in groups of four to a workstation. Each week there was a new “Jamie” recipe and over the course of the program participants learnt to cook dishes from every mealtime i.e. breakfast, lunch, dinner and side dishes. As well as learning cooking skills, the participants were taught knife handling as well as how to bring out the best flavours from fresh foods and how to create dishes without using pre-prepared ingredients. They were also taught how to plan a food budget to help reduce food waste and be more economic in the kitchen. Assessments for the Intervention group were undertaken just prior to their program start date, immediately after their program was completed and 6 months after completion (Fig. 2).

#### Control

The control group’s assessments were undertaken during the six-week period leading up to their participation in the cooking program, thereby representing those who had not undergone the intervention (Fig. 2).

### Primary and secondary outcomes and endpoints

The primary outcome of cooking confidence was evaluated via the theoretical concept of personal beliefs of self-efficacy measured across five areas:

1. Confidence about being able to cook from basic ingredients;
2. Confidence about following a simple recipe;
3. Confidence about preparing and cooking new foods and recipes;
4. Confidence that what is cooked will “turn out” well; and
5. Confidence about tasting foods not eaten before.

This was performed using the same method as described by Flego et al. [42] by measuring cooking confidence as a proxy for self-efficacy. The questions used were based on works by Short (2003) and Barton (2011) and presented on a 5-point Likert confidence scale ranging from ‘not at all’ to extremely’ confident. This was explored in terms of change over time and between different study groups.

The secondary endpoints were measures of dietary intake, physical activity and mobility levels, body composition, anthropometry, blood, urine and faecal biomarkers of systemic, physical and mental health and are further explained in the sections below. These were also explored in terms of change over time and between different study groups.

### Sample size

The sample size calculation in this study was based on the findings of the first published quantitative evaluation of Jamie’s Ministry of Food (JMOF) program into its impact on participants’ cooking confidence [42]. Flego et al. reported an increase of > 0.53 on a 5-point Likert scale across all confidence items, which corresponded to a large Cohen’s effect (d > 0.8), whilst no significant changes were observed in the Control group (all *p* > 0.13). Using G*Power, the required sample for a mixed-model analysis of variance test (ANOVA) design (2 groups, each with 2 time points) to detect (conservatively) a small-medium, within-between, interaction effect (Cohen’s *f* = 0.15), at the 5% level of significance and 80% power was 90, or 45 per group. However, Flego et al. [42] noted that the participant retention rate in the JMOF program at T2 was 55% (694 at T1 and 383 at T2). To account for this and to ensure sufficient participation numbers at the end of T2, the aim was to recruit a minimum of 164 participants with 87 in each time-point (i.e. T1 and T2) of the intervention arm. No power calculation to account for T3 samples size was conducted as the investigation was not aiming to detect a change, rather, whether the effect was maintained at T3.

### Data collection schedule and measures

At all levels of contribution, intervention participants were surveyed at three time points: before the program start (IT1), on program completion (IT2), and 6 months after program completion (IT3). Control participants were surveyed at two time points: 6 weeks prior to program commencement (CT1) and on entry to the program (CT2) (Fig. 1). Computer programmed ‘rules’ automated this process. The ECU SRC ensured consent prior to participation in the study and distributed the online survey assessment using Voxco CATI© Software [47]. The ECU SRC complies with the AMSRS Code of Professional Behaviour and since 2008 SRC has been an ISO20252 standards accredited organisation. All projects undertaken by ECU SRC are conducted in full compliance with the Interviewer Quality Control Australia (IQCA) as recommended by the Australian Market Research Industry and NHMRC/AVCC guidelines on research practices. Postal versions of all documentation were sent to persons who did not have a working email address or access to a computer. The ECU SRC sent participants their online questionnaire 1 week prior to T1, T2 and T3 (for intervention) and if necessary, they received a second reminder email during the week of T1, T2 or T3 as well as a clinic appointment reminder where applicable.

Four levels of contribution were offered to all WA adults registered on the program database (Table 1). The minimum level of contribution, completed by participants at all levels at each time point, consisted of the basic online survey evaluation used in Victoria and NSW [42–45] with the incorporation of additional assessments. These included, quality of life measured using the SF-12® Health Survey [48], wellness using the Subjective Vitality Scale [49, 50] and the Warwick-Edinburgh Mental Well-being Scale (WEMWBS) [51], and physical activity determined using the International Physical Activity Questionnaire (IPAQ) self-administered short-form [52] and the Godin Leisure-Time Exercise Questionnaire [53] (Additional file 1). Those who chose to contribute at level 2 completed the above questionnaire and also attended a clinic appointment at each time point, where they underwent dietary, anthropometric, body composition, blood pressure, timed “Up & Go” [54] and hand grip strength [55] assessments.

#### Dietary assessment

Participants completed a hard copy version of the Dietary Questionnaire for Epidemiological Studies Version 2, food frequency questionnaire (DQES v2, FFQ) developed by Cancer Council Victoria [56] at each time point to assess dietary intake and alcoholic beverage consumption. This questionnaire includes 100 different food items and was designed to assess the usual frequency of dietary intakes over a 12-month period, with 10 frequency response options ranging from ‘never’ to ‘3 or more times per day’. Portion size was calculated using photographs of scaled portions of different food types. Energy (kJ/d) and 31 different intakes of nutrient were calculated by using the NUTTAB95 food composition database [57]. To explore changes after 7 weeks and 6 months for the intervention group and after 5 weeks for the control group the FFQ was re-administered with emphasis on dietary intake in the period being explored. The use of this questionnaire has been validated by Hebden et al. [58] and Petersen et al. [59], however to further explore dietary intake of fruit and vegetables, plasma carotenoids will be measured from participants’ blood samples [58–62]. The use of fresh and healthy ingredients reported by intakes of fruit and vegetables were used to estimate intakes of insoluble fibre, soluble fibre, resistant starch and polyphenols. Dietary fibre, resistant starch and polyphenols are of particular interest due to their interaction with the gut microbiota and their resulting influence on health [62–66].

Similarly, participants completed a short beverage questionnaire that determined the number of cups/glasses of a range of beverages regularly consumed (average over 4 weeks and daily amount), that were used to estimate polyphenol intake from beverages.

#### Anthropometry

Anthropometric measurements included height (m), weight (kg), body mass index (BMI) (kg/m^2^) and waist circumference (cm). Standing height and body mass were measured to the nearest 0.1 cm and 0.1 kg respectively with a SECA 763 digital column scale (SECA Ltd., USA). Waist circumference was measured at the narrowest part of the waist by a Lufkin steel tape measure to the nearest 0.1 cm. All measurements were conducted according to standardised ISAK techniques [67].

#### Body composition

Body Composition was measured using the BOD POD (air displacement plethysmography) (COSMED Asia-Pacific Pty Ltd., Artarmon NSW) The BOD POD uses whole-body densitometry to determine body composition in terms of fat and fat-free mass. All participants who opted for level 2 and above, underwent two tests at each visit lasting approximately 50 s each. The same BOD POD machine was used throughout the study and was calibrated and maintained as per the manufacturer’s recommendations. In some cases, additional measurements of regional and whole-body lean mass and fat mass were derived from a whole-body scan with Dual-Energy X-ray Absorptiometry (DXA) (Hologic 4500A Discovery, Hologic Corp., Waltham, MA, USA). The level of radiation exposure from the DXA scans was negligible (10–30 microSieverts [μSv] in comparison to a flight from Perth to London, approximately 100 μSv). The number of scans conducted in this study were well within the guidelines provided by the manufacturer of the DXA. The scans were conducted by a qualified operator using the same machine each time, however as only a limited number of participants underwent these tests, the results are for reference only.

Due to the inability to relocate the BOD POD machine, participants who attended regional clinics had their body composition, fat mass and fat-free mass measured to the nearest 0.1% using a transportable Impedimed Imp DF50 (Impedimed Ltd., QLD) tetrapolar bioelectrical impedance analyser (BIA). For this procedure participants were asked to lie in a supine position and four electrodes were placed on their hands and feet. A weak electrical current was passed through their body (which they could not feel) and readings from this were used to calculate fat mass, fat-free mass and body water. Reliability of body composition can be influenced by level of hydration. Participants were asked to attend the clinics well hydrated, to refrain from water/fluid ingestion for 1 h prior to the scan and void their bladder.

#### Blood pressure

An Omron IA1B Automated Blood Pressure Device (Omron Healthcare Ltd., Japan) was used to measure participants’ blood pressure. Readings were taken in triplicate with the participant in a calm and relaxed state, in a seated position, on their arm at heart level, at 1-min intervals. All three readings were recorded with the lowest to be used in analysis.

#### Timed “Up & Go” test

Dynamic balance and agility (i.e. functional mobility) were measured using the timed “Up & Go” test [54]. The test quantifies the time it takes to stand up from a seated position in a ‘standard’ chair and walk around a mark placed 3 m from the chair, and then sit back down on the chair. Timing was started as the participant stood up and was stopped once they were re- seated clearly on the chair in an upright position. After familiarisation, one properly executed trial was allowed with time recorded and reported to the nearest 0.01 s.

#### Grip strength

Hand grip strength (kg) was obtained using a Jamar Analogue Hand Dynamometer on the dominant hand [55]. The participant stood with arms by their side and elbow bent at 90 degrees and when instructed to do so, squeezed the dynamometer with as much force as possible. Three trials lasting 3 s each were completed with 15 s rest between them and the maximum value was used for analysis.

#### Measures of biochemistry and urinalysis

Cumulating on level 2 assessments, those who selected the third level of contribution also provided blood and urine samples for biochemical analysis at each time point. Blood and urine samples were collected by trained phlebotomists in the morning after an overnight fast. The venous blood samples were centrifuged and processed within 30 min after collection to separate plasma and serum. These were then aliquoted into 1.5 or 2 mL vials and stored at − 80 degrees until analysis. Biochemical variables related to cardiovascular, liver, cognitive and musculoskeletal disease such as fasting blood lipids, glucose and high sensitivity C-reactive protein will be measured by routine laboratory methods. Carotenoid concentration (α-carotene, β-carotene, lycopene, β-cryptoxanthin, lutein) will be analysed as objective indicators of fruit and vegetable intake [68]. Participants gave their permission for future analyses that include the following but are dependent on available funding; advanced glycation end products (IgG *N*-glycans) measured to assess early signs of health risks such as hypertension, inflammatory diseases and cardiovascular diseases [69]; serotonin and lipopolysaccharide (LPS) as biomarkers of mental and gut health [70, 71].

#### Human faecal sample collection, processing and analysis

The fourth and maximum level of contribution incorporated the additional provision of a 24-h stool sample at each time point. Instructions and equipment were provided for the collection of stools from all bowel motions over a 24 h period, if more than one stool sample was collected, they were homogenised as individual samples and then pooled and homogenised again. This was done to provide a more accurate, homogenous measure of the gut microbiota and gut metabolites. Participants were given a portable cooler bag and frozen icepacks to store the stool samples over the 24 h collection period and to return their sample(s) to the clinic. Once received the frozen stool samples were weighed and stored at − 80 degrees. Stool samples were thawed in a fridge (max 4 °C) and homogenised before being aliquoted for various analyses. Stool aliquots for short chain fatty acids (SCFA), bile acids and microbial analysis remain at − 80 °C until their analysis.

Microbial analyses will be performed at the WA Human Microbiome Collaboration Centre, Perth WA. DNA will be extracted using the QIAamp PowerFecal DNA kit (Qiagen) using Qiacube extraction platform. Bacterial signatures are generated using the Illumina MiSeq platform using uniquely barcoded 16S rRNA gene primers (515–806(V4)), following polymerase chain reaction (PCR) inhibition assessment of each DNA extract. PCR-free ligation protocol is thereafter deployed for library building. Samples will be sequenced to a depth of minimum 50,000 reads, which is sufficient to identify microbes to a genus/species level. Quality control and mock community samples are included in the analysis from sample collection to sequence analysis. Sequence read quality is initially assessed with FastQC before demultiplexing and pre-processing by GHAPv2, an in-house tool. Cutadapt [72] is used for removal of all non-biological sequences. DADA2 [73] is then used for quality filtering, error correction and amplicon sequence variants (ASVs) picking. A trained naïve bayes classifier then assigns the ASV’s to genus/species against a curated database of microbial reference sequences such as the RDP [74] or Genome Taxonomy Database [75].

Measures of dietary fibre and protein fermentation in the stool samples will be determined from short chain fatty acids (SCFA) using gas chromatography mass spectrometry at ECU. SCFA in faecal material will be extracted into an acidified aqueous methanol solution and the SCFA separated by gas chromatography using a fatty acid column (Zebron ZB-FFAP 30 m × 0.53 mm × 1 μm supplied by Phenomenex). The SCFA will be detected using mass spectrometry and their concentrations determined by the method of internal standards. Isotopically internal standards will be spiked into the extraction solution.

As high fat diets have been associated with poorer health outcomes, in addition to blood lipids, high performance liquid chromatography in tandem with mass spectrometry will be used to analyse faecal bile acids. Beyond their role of facilitating fat digestion, recent research discoveries have emphasised the importance of bile acid metabolism as a regulator of human physiology and central to overall health [76, 77] and disruptions to BA processing are associated with the onset of chronic disease. The target bile acids will be extracted from the faecal material by solid phase extraction and then injected into a C18 column. The separated bile acids will be identified and quantified by mass spectrometry using a method developed by ECU and adapted from [78, 79]. The method of internal standards will be adopted to correct for matrix effects.

**Fig. 3:**
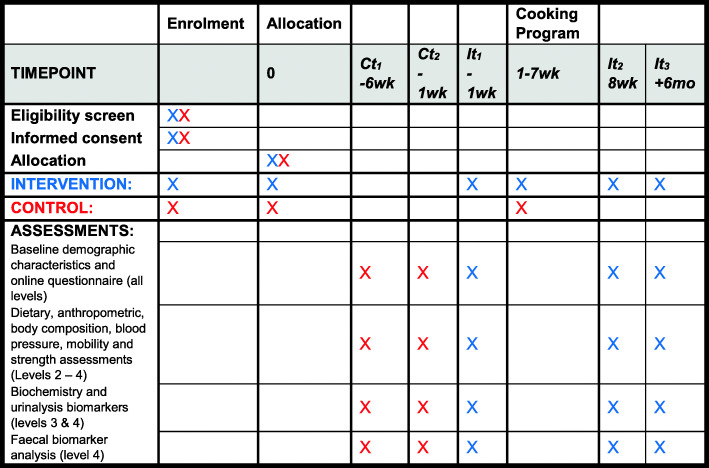
SPIRIT figure for study protocols

The SPIRIT figure for study protocols is presented in Fig. 3 and the 33 item SPIRIT checklist in Additional file 2 (separate upload) [80, 81].

### Data management

Data management (collection, recording, and storage) complies with the rules of ECU Human Research Ethics Committee (HREC) and is reported on annually (ID 15362:Newton). All data has been de-identified and stored according to HREC policy in access restricted, secure repositories.

### Statistical methods

The computer software package IBM SPSS Statistics for Windows version 25.0 (Armonk, NY: IBM Corp) will be used to access the data set and perform the statistical analyses. For the basic online evaluation questionnaire, general descriptive analyses including frequency data with appropriate plots and cross-tabulations will be used to explore and summarise outcome variables. Continuous demographic and baseline characteristics will be summarised using standard statistics (mean and standard deviation) and non-parametric statistics (medians and inter-quartile ranges) where appropriate. Independent *t*-test for normally distributed data, and Mann-Whitney U test for non-normal data, will be used to assess differences between the groups. Frequencies and percentages will be reported for categorical variables and Chi-square test will be used to test for differences in proportion between the groups. The magnitude of change both within and between the intervention and control groups will be assessed using mixed-model ANOVA. Furthermore, changes within the intervention group will be assessed across three time points IT1, IT2 and IT3 using repeated-measures ANOVA. To account for multiple outcomes, the Benjamini-Hochberg correction (i.e. false discovery rate) will be applied to the raw *p*-values. Participants with missing value for the variables of interest (either outcome or predictor) will be removed from the repeated-measures ANOVA models. Specific demographic factors of interest (including but not limited to, gender, age, education, BMI and employment) will be adjusted for in all models. Statistical significance will be indicated at the 10, 5 and 1% level. Cohen’s effect sizes will be provided as a measure of practical/clinical significance and to assist in the interpretation of said ‘statistically significant’ results.

To interpret gut microbiota diversity and to analyse the microbial composition of the gut microbiota, multivariate analysis using a combination of R and PRIMER7 and Permanova+ (PRIMER-E, Plymouth) [82] will be conducted. Principal Coordinates Analysis (PCoA) will be deployed to visualise findings. Distance-based linear models (DISTLM) and distance-based redundancy analysis (dbRDA) will be used to integrate microbiome findings with other relevant data (i.e. dietary intake) that help explain the relationship between the microbiome findings and other outcomes.

## Discussion

It is well reported that an increased emphasis on families and individuals to change their dietary patterns and lifestyle can have significant impact on weight reduction and overall health [29, 83–87]. Family dinners are have been found to be a measurable signature of these social exchanges in the home that support socioemotional development and mental health [88]. A 2017 study reported an association between increased frequency of family dinners and lower rates of obesity that was pronounced for those whose meals were home-cooked [89]. Indeed, the consensus statement of the International Society for Nutritional Psychiatry Research (ISNPR) stresses the importance of diet and nutrition as a critical determinant of mental and physical health [90]. The collaboration of academics advocates for future public health interventions to stimulate a significant change in eating habits back to more traditional wholefood diets.

Yet, research in WA suggests that the demands on families [91–93], together with fatigue [93], lack of time, family/social support and overall motivation [91, 93, 94], impede prioritisation of a healthy diet and lifestyle [95].

The JMOF mobile kitchen has been delivering seven-week cooking programs to the community teaching basic cooking skills aimed at improving participants’ knowledge and ability to purchase, prepare and cook healthier meals at home. This study protocol describes the recruitment of willing participants and the assessment methods involved in an evaluation of the program’s effect on their cooking confidence, dietary intakes, physical and mental health and wellbeing. The study extends on previous, similar studies in that it includes collection and analysis of biomarkers of both metabolic and gut health. The study will provide valuable insight into the effectiveness of food literacy cooking programs and how improvements to cooking confidence and skills can contribute to healthier outcomes.

Strengths of the study include the extensive range of data collected that comprise; self-reported cooking confidence and self-efficacy and measures of physical and mental health and well-being. In addition, data has been collected on self-reported dietary intake; measures of health status from anthropometry, body composition, blood pressure, agility and grip strength, blood, urine and faecal biomarkers. The study has also collected longitudinal data to explore sustainability. Findings will support future public health cooking program initiatives aimed at improving food literacy, self-efficacy and physical and mental health. It is also novel in that it will help to identify mechanistic associations between dietary intake and interactions between human gut microbiota, their metabolites and their interplay with host metabolism in both physiological and mental health.

There is burgeoning evidence reporting the beneficial associations between the human gut-microbiome and the key microbes and their metabolites that are associated with health [18, 19]. Diets higher in protein and fats and lacking in fermentable substrates for the production of beneficial SCFA have increasingly been linked to poorer physical and mental health outcomes [65, 96–98] and the presence of microbial profiles that are more often associated with disease [76]. The inclusion of faecal bile acid analysis will enable investigation of their role in human metabolism beyond their core function of assisting with dietary fat digestion and absorption. Faecal bile acid composition is of great interest when studying the role of diet and gut microbiota in health and disease, due to the bi-directional interplay between gut bacteria and bile acid and cholesterol metabolism that leads to implications on host physiology [76, 99].

The study is limited due to the confines of the study design that prevented true randomisation. According to the Stages of Change theory [100], as the study participants were selected from the wait-list and had already committed to the program, they would have transitioned from the pre-contemplation to the change/action stage. Therefore the act of registering for the program could potentially instigate behaviour change, which may result in positive change within both groups [101]. As a consequence, the control group may not truly reflect the general population. The standard evaluation used the tool created for previous Australian cooking programs that were run in Victoria and NSW [42–44], however this study extended data collection to include more detailed assessments of physical and mental health as described in this protocol. The DQES vs 2 FFQ is a reflection of intake over 12 months, however it has been validated to detect change over a shorter timeframe in similar studies [58, 59].

The study’s strengths include the extensive data collected that will provide valuable insight to the effects that a basic cooking program incur within a healthy population of Australian adults. Findings will inform on the complex interaction between self-efficacy, dietary intake, gut-microbiota and biomarkers of physiological health and well-being, that may support the employment of basic cooking programs as a tool for future health promotion interventions.

## Supplementary information

**Additional file 1.** Additional information [48–53, 102]

**Additional file 2.** SPIRIT checklist

## Data Availability

The datasets generated and/or analysed during the current study are not publicly available due to there being a consulting services deed agreement between ECU and The Good Foundation that prohibits the disclosure of data for this study. Data are however available from the authors upon reasonable request and with permission of The Good Foundation.

## Abbreviations

ANZCTR: Australian and New Zealand Clinical Trials Registry
ABS: Australian Bureau of Statistics
DF: dietary fibre
JMOF: Jamie’s Ministry of Food
WA: Western Australia
TGF: The Good Foundation
ECU: Edith Cowan University
HREC: Human Research Ethics Committee
SRC: Survey Research Centre
IQCA: Interviewer Quality Control Australia
ANOVA: analysis of variance test
WEMWBS: Warwick Edinburgh Mental Well Being Scale
IPAQ: International Physical Activity Questionnaire
DQES vs 2 FFQ: Dietary Questionnaire for Epidemiological Studies Version 2, food frequency questionnaire
BMI: body mass index
DXA: Dual-Energy X-ray Absorptiometry
BIA: bioelectrical impedance analyser
LPS: lipopolysaccharide
PCR: polymerase chain reaction
ASVs: amplicon sequence variants
SCFA: short chain fatty acids
PCoA: Principal Coordinates Analysis
DISTLM: distance-based linear models
dbRDA: distance-based redundancy analysis
NaOH: Sodium Hydroxide
