# Supplementary material for: The study protocol for a pseudo-randomised pre-post designed controlled intervention trial to study the effects of a 7-week cooking program on self-efficacy and biomarkers of health: the ECU lifestyle and biomarkers get connected study (ECULABJMOF) including the Jamie’s Ministry of Food WA participant experience
Source: BMC Public Health. 2020 Jun 30;20:1037. doi: 10.1186/s12889-020-09124-3 (PMC7325354; doi:10.1186/s12889-020-09124-3)
Supplement: Supplementary file 1 — Additional file 1. Additional information [48–53, 102] [file 12889_2020_9124_MOESM1_ESM.docx]

**Additional file 1.**

**Quality of life**

Quality of life was assessed using the SF-12® Health Survey (49) which includes 12 questions from the SF-36® Health Survey (Version 1) (103). These include: 2 questions concerning physical functioning; 2 questions on role limitations because of physical health problems; 1 question on bodily pain; 1 question on general health perceptions; 1 question on vitality (energy/fatigue); 1 question on social functioning; 2 questions on role limitations because of emotional problems; and 2 questions on general mental health (psychological distress and psychological well-being).

**Wellness**

The adapted 6-item version of the Subjective Vitality Scale was used to measure the state of feeling alive and alert and having energy. Those high in subjective vitality are reported to be alert, energized, and vital (50, 51).

The Warwick-Edinburgh Mental Well-being Scale (WEMWBS) (52) is a short 14 item psychometric measure of mental well-being. The measure has a focus on positive aspects of mental health and no population-based ceiling effects. It has a role in evaluation of mental health promotion initiatives due to the sensitivity of the scale to change.

**Physical activity**

Physical activity level was assessed by the International Physical Activity Questionnaire (IPAQ) self-administered short-form (53) which has a total of 7 questions. The IPAQ assessed vigorous and moderate activities that were undertaken in the previous 7 days recorded as the equivalent minutes of exercise per week. Self-reported physical activity was also assessed by the Godin Leisure-Time Exercise Questionnaire (2 questions) (54).
